# Cyto- and Myelo-Architecture of the Amygdaloid Complex of the Common Marmoset Monkey (*Callithrix jacchus*)

**DOI:** 10.3389/fnana.2019.00036

**Published:** 2019-03-27

**Authors:** Paulo Leonardo Araújo Góis Morais, María García-Amado, Ruthnaldo Rodrigues Melo Lima, Angélica Córdoba-Claros, Jeferson Souza Cavalcante, Francisco Clascá, Expedito Silva Nascimento

**Affiliations:** ^1^Department of Morphology, Universidade Federal do Rio Grande do Norte, Natal, Brazil; ^2^Department of Anatomy & Neuroscience, School of Medicine, Autonoma de Madrid University, Madrid, Spain; ^3^Department of Physiology, Universidade Federal do Rio Grande do Norte, Natal, Brazil

**Keywords:** marmoset *Callithrix jacchus*, amygdaloid complex, tractography, myelin staining, nuclear division

## Abstract

The amygdaloid complex (AC) is a heterogeneous aggregate of nuclei located in the rostromedial region of the temporal lobe. In addition to being partly connected among themselves, the AC nuclei are strongly interconnected with the cerebral cortex, striatum, basal forebrain, hypothalamus and brainstem. Animal and human functional studies have established that the AC is a central hub of the neuronal networks supporting emotional responsivity, particularly its negative/aversive components. Dysfunction of AC circuits in humans has been implicated in anxiety, depression, schizophrenia and bipolar disorder. The small New-World marmoset monkey *(Callithrix jacchus)* has recently become a key model for neuroscience research. However, the nuclear and fiber tract organization of marmoset AC has not been examined in detail. Thus, the extent to which it can be compared to the AC of Old-World (human and macaque) primates is yet unclear. Here, using Nissl and acetylcholinesterase (AChE) histochemical stains as a reference, we analyzed the cytoarchitecture and nuclear parcellation of the marmoset AC. In addition, given the increasing relevance of tractographic localization for high-resolution *in vivo* imaging studies in non-human primates, we also identified the myelin fiber tracts present within and around the AC as revealed by the Gallyas method. The present study provides a detailed atlas of marmoset AC. Moreover, it reveals that, despite phylogenetic distance and brain size differences, every nucleus and myelinated axon bundle described in human and macaque studies can be confidently recognized in marmosets.

## Introduction

The primate amygdaloid complex (AC) is a heterogeneous cluster of gray matter nuclei and fiber tracts in the anteromedial temporal region of the cerebral hemisphere. Developmental studies have shown that AC cells have remarkably diverse neuroepithelial origins. The cells in the basal-lateral nuclei of AC derive from lateral and ventral pallial neuroepithelial matrices (Medina et al., 2004), those in the central nucleus (Ce) derive from the subpallium, and medial (Me) and cortical nuclei cells derive from the ventral pallium, subpallium and even from some areas outside the telencephalon (García-López et al., 2008). Accordingly, the extrinsic connections of these three main nuclear groups are disparate; basal-lateral group nuclei are mainly connected with sensory, association and limbic areas of the neocortex, thalamus and striatum, Me and cortical nuclei are linked with olfactory bulb and basal forebrain structures, and the Ce is associated with hypothalamus and brainstem structures. Moreover, while direct connections between nuclei of the same group are abundant, those between nuclei of different groups are quite limited (Swanson and Petrovich, 1998).

A wealth of functional studies in different mammals, including primates, have revealed that the AC is a central hub in the neuronal networks creating emotional responsivity, particularly its negative/aversive components. However, consistently with their heterogeneous developmental origin and connections, the various AC nuclei are preferentially involved in specific responses. For example, studies in rats have shown that the innate and acquired aspects of fear responses to threatening stimuli depend on specific AC circuits (Canteras et al., 2012; Gross and Canteras, 2012; Bindi et al., 2018). Similarly, studies in primates indicate that the basolateral nuclei are related to the acquisition of conditioned fear, the Me are involved in processing threat-related odors and the Ce may act as an interface between the rest of the AC and hypothalamic and brainstem areas controlling emotional/visceral responses (Fox et al., 2015, 2018; Wellman et al., 2016).

Because of its small size and fast breeding rate in captivity, the common marmoset monkey (*Callithrix jacchus*), a species native from Northeastern Brazil, has in recent years become a key non-human primate model species for biomedical and neuroscience research (Mitchell and Leopold, 2015; Riesche et al., 2018; Veyres et al., 2018), as well as the focus of Japan’s Brain/MINDS brain mapping project (Okano et al., 2016,^1^). On the other hand, comparison of marmoset brain circuitry and function with that of the extensively studied Old World macaque monkeys or humans is not straightforward, not only because differences in size and the absence of most cortical sulci in marmosets, but also because paleontological and genetic studies consistently indicate that the marmoset (parvorder *Platyrrhini*) and the macaque/human lineages (parvorder *Catarrhini*) have been evolving independently for at least 30 million years (Steiper and Young, 2006; Hodgson et al., 2009).

Some data on the marmoset AC histology can be derived from published studies focused on other brain regions (Roberts et al., 2007; Carlo et al., 2009; Burman et al., 2011; Paxinos et al., 2012). No study, however, has to date specifically investigated the marmoset AC nuclear organization with a detail comparable to that of published studies on macaque and human amygdala. Thus, it remains unclear to which extent the same nuclear subdivisions described in macaque and human ACs can be recognized in marmosets. Moreover, there is virtually no information about the white matter tracts around and within the marmoset AC; this information is relevant for studies using new tractographic imaging techniques *in vivo* (Okano et al., 2016; Schaeffer et al., 2017).

Here, we report a detailed delineation of AC nuclei boundaries and myelinated fiber tracts based on the comparison of alternate serial sections stained for Nissl substance, acetylcholinesterase (AChE) and myelin. Our results show that, despite some differences in relative size, the nuclear subdivisions and fiber tracts of marmoset AC closely resemble those described in the macaque and human AC.

## Materials and Methods

### Animals

Serial brain sections taken from four adult marmosets (two females and two males, aged 1.5–4 years and weighing 250–390 g), were used for the analysis described here. The animals were bred at the Primatology Center of the Federal University of Rio Grande do Norte (UFRN, Natal, Brazil), and were housed under natural lighting, temperature and humidity conditions, with food and water *ad libitum*. This study was carried out in accordance with the recommendations of “the UFRN Animal Experimentation Ethics Committee (024.028/2017)”. The protocol was approved by the “UFRN Animal Experimentation Ethics Committee (024.028/2017)”. As a part of a separate study, 2 weeks before sacrifice the same animals had received, under isoflurane anesthesia, tracer deposits (Fast blue, Diamidino Yellow) in dorsal regions of the cerebral cortex, through small craniotomies that left the temporal region intact. After the surgery, animals had received a single i.m. dose (0.8 mg/100 g) of the antibiotic cefavezin (Convenia, Pfizer) and the analgesic buprenorphine 0.1 mg/100 g, (Buprenex, Reckitt Benckiser Healthcare, UK).

### Tissue Fixation and Histological Procedures

Animals were perfused through the left ventricle with saline (0.9%, 5 min), then 4% paraformaldehyde + 0.2% glutaraldehyde in PB (0.1 M, pH 7.4, 4°C, 30 min) and finally 4% paraformaldehyde + 15% sucrose in PB (0.1 M, pH 7.4, 15 min). The calvarium and duramater were then excised, and the head was positioned in the stereotaxic frame to cut the brain in tissue blocks precisely aligned to the Horsley-Clarke coronal plane (Paxinos et al., 2012). Tissue blocks were then removed from the skull, postfixed for 24 h in the last perfusion solution, and then soaked in 30% sucrose in PB (0.1 M, 4°C) until they sank.

From each tissue block, six parallel series of 40 μm-thick coronal brain sections were obtained on a Leica freezing microtome. Sections were collected in PB (0.1 M, 4°C). Three of the section series were used for the present study. The first series was mounted on glass slides, air dried, and then stained with Cresyl violet, differentiated first in graded alcohols and then in 96% ethanol + 2% glacial acetic acid, dehydrated, defatted in xylene, and coverslipped with Depex. The second series underwent a standard histochemical protocol to reveal AChE activity (Geneser-Jensen and Blackstad, 1971) before being mounted and coverslipped as above. The third series was stained, free-floating, to reveal myelin with the Gallyas physical development method (Gallyas, 1979), and subsequently mounted and coverslipped as above.

### Microscope and Image Analysis

Eigth (left and rigth-side) ACs were analyzed. For each coronal level, three adjacent 40 μm sections, each one stained for Nissl substance, AChE or myelin were examined. A 120 μm-wide gap separated each sampled level from the following level. Tissue sections were imaged with a Nikon Eclipse 600 microscope under 4×–20× bright-field optics. The motorized microscope stage controlled by the NIS Elements Nikon software was used to combine multiple field images taken with the 10× objective into panoramic views of the entire AC complex and surrounding structures. Using Canvas XV software (ACD Systems, Victoria, BC, Canada), the nuclei and fiber bundle boundaries recognizable with each technique were first drawn on the image files. Using blood vessels and other anatomical features for precise alignment, the drawings were finally overlaid and combined in a single final diagram.

## Results

The staining patterns produced by the three techniques we used were consistent among cases. In the description below, we first provide an overview of the location and general appearance of the marmoset AC as it appears in a rostral-to-caudal sequence of coronal sections. Then, we describe each nucleus and constituent nuclear subdivisions in detail. Finally, we discuss our observations regarding the various fiber bundles around and within the AC, as seen with myelin stains.

### Overall Appearance of the AC as Seen in a Rostral-to-Caudal Series of Coronal Sections

The marmoset AC is a round/oblong mass of gray matter nuclei and fiber tracts, roughly about 4 mm in diameter, situated in the anteromedial zone of the temporal pole, between interaural coronal planes +10.5 and +6.8 (Paxinos et al., 2012). Most of the mass of the AC is buried deep within the cortical parenchyma; some AC nuclei reach the pia of the anteromedial surface of the temporal lobe, while others are more deeply located, some of them protruding on the lateral wall of the lateral ventricle temporal horn. Compared to mammals with a less expansive temporal cortex such as rodents, the overall position of AC nuclei appears medially “rotated” about 90°. This position is roughly the same than that of AC in the macaque brain (Freese and Amaral, 2005). The human AC shows an even more pronounced degree of medial rotation (García-Amado and Prensa, 2012). Cell somata situated deep within the AC are more or less evenly distributed, while those in the subpially-located AC regions usually show a coarse layering, parallel to the pia, which is continuous with that of the adjacent entorhinal (E) or subicular cortices.

For consistency across species, for our description, we follow the nomenclature proposed for the macaque AC by Price et al. (1987) and Pitkänen and Amaral (1998) that has also been applied to descriptions of the human AC (García-Amado and Prensa, 2012). Thus, we subdivide the AC in four main nuclear groups: (a) a Superficial group [which includes the Cortical nuclei, the Me, the Periamygdaloid cortex (PAC), the nucleus of the lateral olfactory tract (NLOT)]; (b) a Deep group (encompassing the Basal (B), Lateral (L), Accessory Basal (AB) and paralaminar (Pal) nuclei); (c) the Ce; and (d) the Intercalated nuclei (I).

In addition, we distinguish two “transitional” areas between the AC and adjacent structures: (a) the anterior amygdaloid area (AAA), which is rostromedially continuous with the substantia innominata (SI; this region is often referred to as part of the “extended amygdala,” de Olmos and Heimer, 1999); and (b) the amygdalohippocampal area (AHI), which is caudomedially continuous with the hippocampus (Hip).

**Figure 1:**
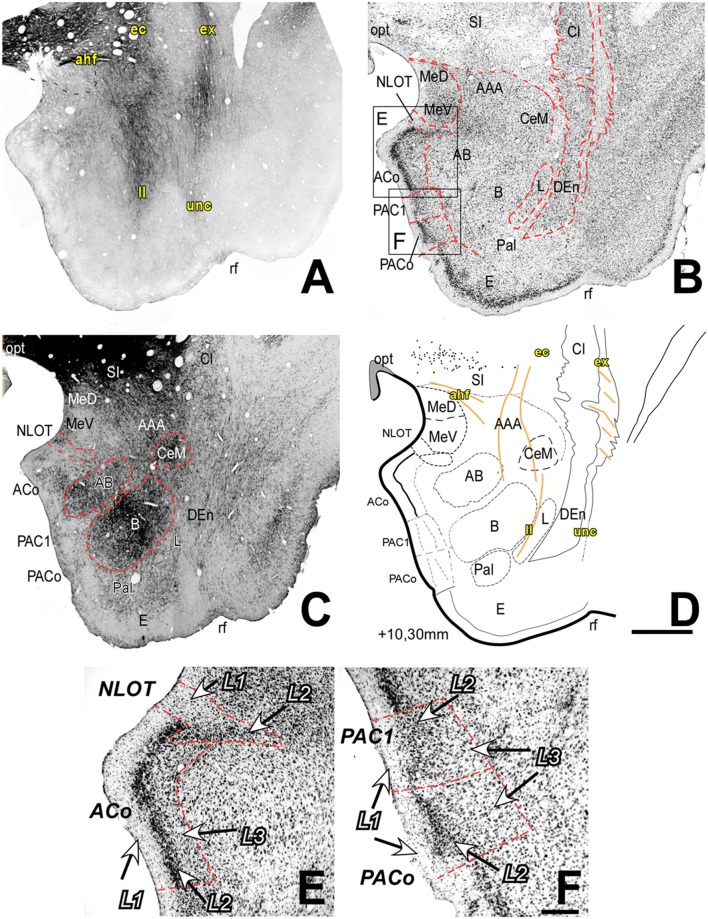
Myelo- and cytoarchitecture of the amygdaloid complex (AC), level 1 (Interaural 10.30 mm). Series of adjacent coronal sections stained for Gallyas **(A)**, Nissl **(B)** and acetylcholinesterase (AChE) **(C)** and the corresponding drawing showing in black the territories of the AC and in orange the myelinic bundles schematically **(D)**. At this level, it is already possible to identify the deep nuclei, and some of the superficial nuclei and myelin tracts located around and within the nuclei and helping in their delimitation. Unambiguous nuclear boundaries are drawn with a red dashed line in **(B,C)**. **(C)** Note the intense staining of AChE in the Basal nucleus (B) and, although heterogeneous, in the accessory basal (AB); CeM showed a markedly high AChE staining level. Panels **(E,F)** are high magnification bright-field photomicrographs from Nissl-stained sections in the superficial areas of the AC [corresponding to the black squares in **(B)**]; the layers are indicated in each region. **(E)** The anterior cortical nucleus (ACo) contains a densely stained and cell-packed L2; the nucleus of the lateral olfactory tract (NLOT) shows a large L1. **(F)** PACo (inferior) and periamygdaloid cortex (PAC) 1 (superior). Note the different cell compaction of L2 between these two regions. Scale: 1 mm in **(A–D)** and 200 μm in **(E,F)**.

In the most anterior AC coronal section levels (~AP +10.30 mm, Figure 1), the Superficial nuclei group is most apparent: the Me occupy a dorsal position, sandwiched between the optic tract (opt) and AAA, while the Cortical nuclei cover the anteromedial pial surface of the temporal pole. The most anterior cells of the Ce can be seen interspersed among the fibers of the external capsule (ec), lateral to the AAA, and dorsolaterally wrapped by the mass of myelinated fibers of the posterior limb of the anterior commissure (acp). Likewise, the most anterior cell groups from the three large nuclei of the Deep group (L, B and AB nuclei) are already visible at this coronal section level. Laterally to the ec and the lateral medullary lamina (ll), the Claustrum (Cl) and the dorsal subdivision of the Endopiriform nucleus (DEn) form a continuous cell band (Smith et al., 2018). At levels rostral to AP +10.50 mm, the AC and amygdaloid capsule (amc) disappear, and the prepyriform cortex covers the rostral tip of the temporal pole (not shown).

**Figure 2:**
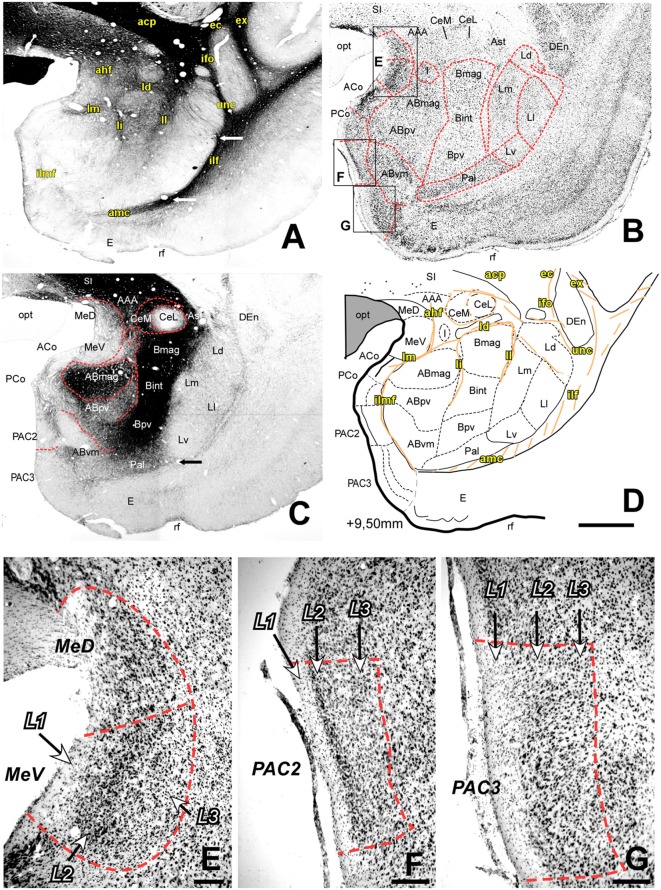
Myelo- and cytoarchitecture, level 2 (Interaural 9.50 mm). Series of adjacent coronal sections stained for Gallyas **(A)**, Nissl **(B)** and AChE **(C)** and the corresponding drawing showing in black the territories of the AC and in orange the myelinic bundles schematically **(D)**. Unambiguous nuclear boundaries are drawn with a red dashed line. The divisions of the Ce showed huge differences in AChE staining intensity at this level **(C)**. In **(A)**, white arrows indicate the boundaries between the L and the Pal (bottom) and between the Ld and Ll subdivisions (top). Also, interesting is the presence of highly AChE stained I between the Ce and AB **(C,D)**. Myelin fibers help in the delimitation of the Ce and are abundant in the Bmag and Bint **(A)**. Panels **(E–G)** are high magnification bright-field photomicrographs of Nissl-stained sections in different portions of the superficial areas of the AC [corresponding to black squares in **(B)**]; the layers are indicated in each region. In **(E)**, the rostral level of the Me shows three obvious layers in the ventral division which were not visible in the dorsal division. At this level, there are two subdivisions of the PAC, each of them showing a characteristic layer pattern: **(F)** In the PAC2 subdivision, L2 is thin and has densely packed cells; **(G)** PAC 3 has a large L2 formed by big and highly stained cells. Scale: 1 mm in **(A–D)** and 200 μm in **(E–G)**.

**Figure 3:**
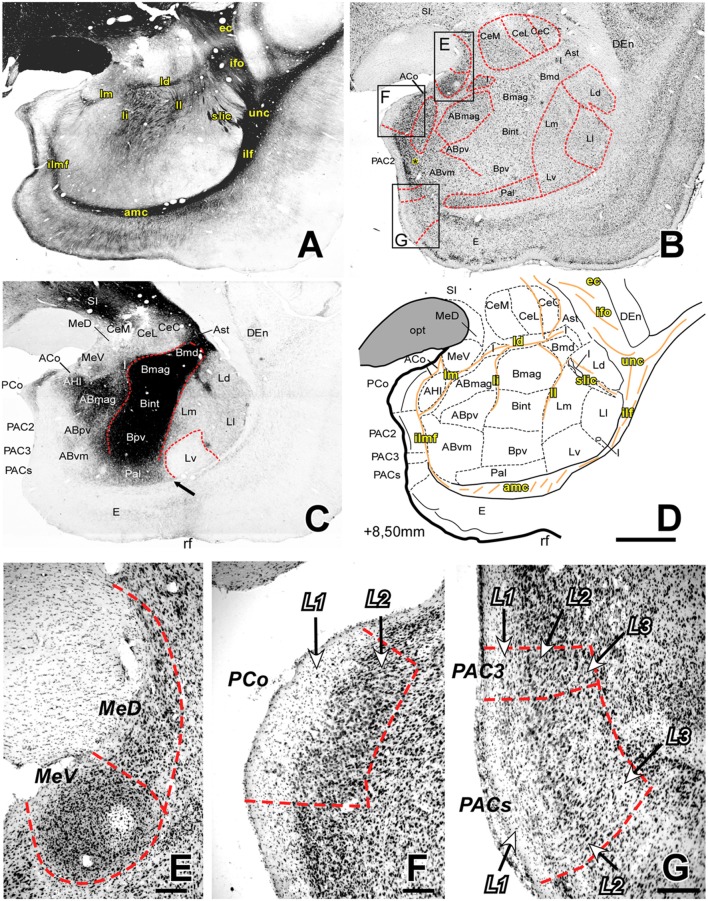
Myelo- and cytoarchitecture, level 3 (Interaural 8.50 mm). Series of adjacent coronal sections stained for Gallyas **(A)**, Nissl **(B)** and AChE **(C)** and the corresponding drawing showing in black the territories of the AC and in orange the myelinic bundles schematically **(D)**. Unambiguous nuclear boundaries are drawn with a red dashed line. The divisions of the Ce showed no difference in AChE at this level **(C)**. The presence of heavily AChE stained I within the L, separating its subdivisions **(C,D)** is also distinctive in this level. Myelin fibers clearly demarcate the ventral border of the Ce and are very abundant in the B **(A)**. Panels **(E–G)** are high magnification bright-field photomicrographs from Nissl-stained sections in different portions of the superficial areas of the AC (corresponding to the black squares in **B**); the layers are indicated in each region. In **(E)**, the most caudal level of the Me, the MeV has lost the layered pattern and contains highly compacted cells; **(F)** posterior cortical nucleus (PCo) is characterized by only two layers. **(G)** Two of the three PAC subdivisions present at this level are shown: PAC 3 (superior) and PACs (inferior), both containing lightly stained neurons in L1. Scale: 1 mm in **(A–D)** and 200 μm in **(E–G)**. The asterisk in panel B is located in L3 of PAC2, which contains more scattered Nissl-stained cells than L2.

**Figure 4:**
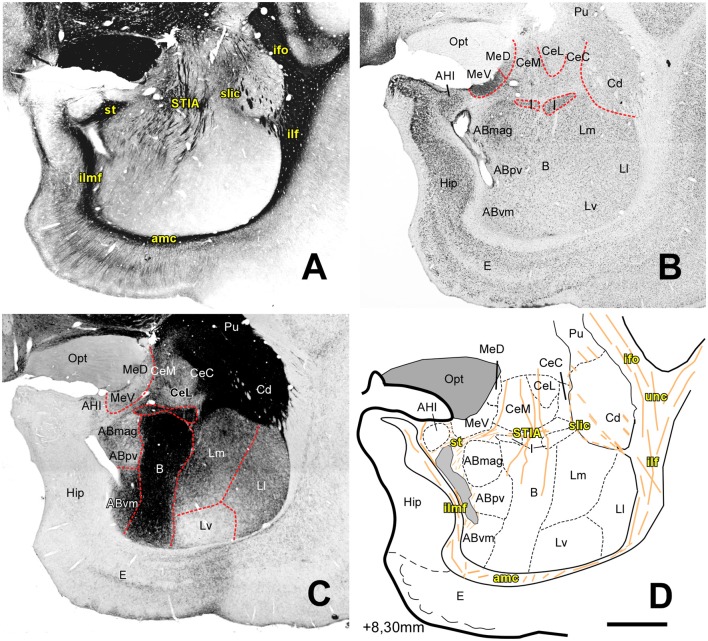
Myelo- and cytoarchitecture, level 4 (Interaural 8.30 mm). Series of adjacent coronal sections stained for Gallyas **(A)**, Nissl **(B)** and AChE **(C)** and the corresponding drawing showing in black the territories of the AC and in orange the myelinic bundles schematically **(D)**. Unambiguous nuclear boundaries are drawn with a red dashed line. At this level, the Hip appears medial to the AC, the cytoarchitectonic profile of the B and AB nuclei becomes diffuse, and the Ld subdivision is not distinguishable in the L. The divisions of the Ce show differences in the AChE staining at this level, with the capsular subdivision being more intensely stained **(C)**. The location of the I coincides with an intense myelin stained region and with an appearance of the st and its intraamygdaloid component (STIA). Scale: 1 mm.

Coronal sections taken at about AP +9.80 to +8.00 mm (Figures 2, 3) make all the major cellular and fiber AC components fully apparent. At these levels, most of the extent is occupied by the large masses of the Deep group nuclei, which display sharply different cytoarchitectures and AChE staining patterns between them (see below). The Cortical nuclei of the Superficial group disappear caudally to level AP +8.30 mm (Figure 4), as they are replaced, at the same position, by the rostral part of the subiculum and Ammon’s horn. In contrast, the Me remain visible, although they become progressively smaller at more caudal section levels. The oval cell mass of the Ce is sharply delineated from the Deep nuclei by a narrow dense band of myelinated fibers, the dorsal medullary lamina (ld). The I appear as dense clumps of small cells situated in between some of the larger nuclei.

**Figure 5:**
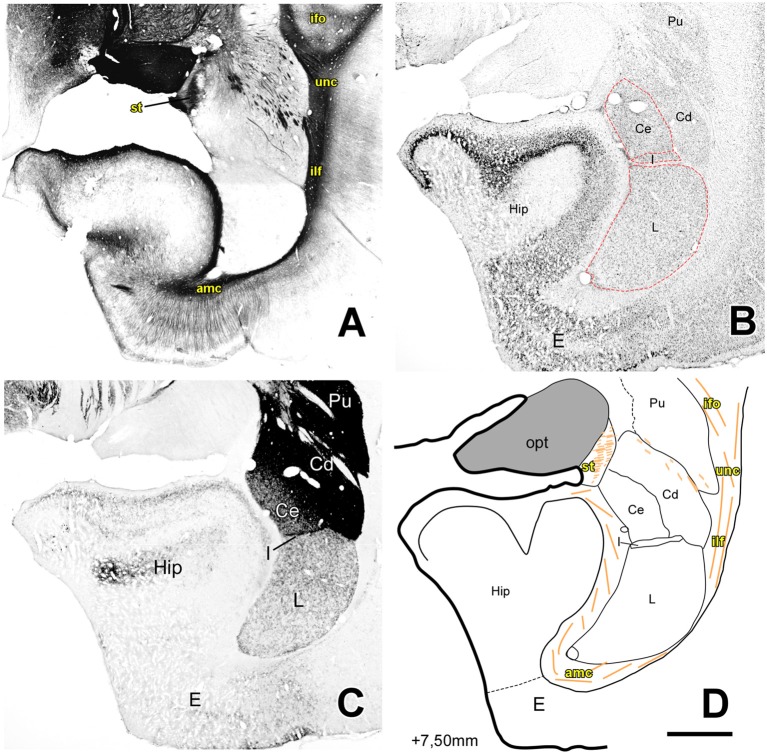
Myelo- and cytoarchitecture, level 5 (Interaural 7.50 mm). Series of adjacent coronal sections stained for Gallyas **(A)**, Nissl **(B)** and AChE **(C)** and the corresponding drawing showing in black the territories of the AC and in orange the myelinic bundles schematically **(D)**. Unambiguous nuclear boundaries are drawn with a red dashed line. At this level, the Hip shows a bigger volume than the AC and only the L, Ce, I and Me nuclei were present. At this level, STIA is adjacent to the opt and a strongly AChE stained I separated the Me and Ce nuclei from the L. Scale: 1 mm.

At progressively more caudal AC levels (Figures 4, 5; AP +8.0–6.80) the Ce and Me are replaced by the rostromedial tip of the temporal horn of the lateral cerebral ventricle (around AP +7.30). Some nuclei of the Deep group (AB and B) also disappear at this coronal level, replaced by the expanding mass of the Ammon’s horn. Only the L extends up to the caudal end of AC. This caudal zone of the L is sandwiched in between the lateral ventricle wall and the caudate nucleus (Cd), but always clearly delineated from the latter by their different AChE staining pattern, as well as by the presence of a thin band of myelinated fibers.

### Nuclei Subdivisions Based on Cytoarchitectonics and AChE Staining Patterns

#### The Superficial Nuclei

These AC nuclei are structures located in the medial surface of the temporal lobe, their most prominent characteristic is their layers, particularly layer 2 (L2), which varies in thickness, cell aggregation and cell size. We distinguished five superficial nuclei or structures: the Me, the anterior cortical nucleus (ACo), the posterior cortical nucleus (PCo), the NLOT and the PAC.

##### The Medial Nucleus (Me)

The Me has a superficial position and an irregular shape along the rostrocaudal axis of the AC (Figures 1D, 2D, 3D, 4D). The medial medullary lamina (lm) separates it from the AB (Figures 2A, 3A) and from the AHI (Figure 3A). We identified two subdivisions on the basis of the cytoarchitecture and AChE activity: a dorsal subdivision (MeD) characterized by sparse neurons, and a ventral division (MeV) with bigger and more densely packed neurons (Figures 2E, 3E) and stronger AChE staining at rostral levels (Figures 1C, 2C) but lower at caudal portions of the AC (Figures 3C, 4C). In the rostral half of the nucleus, three cell layers are distinguishable in the MeV (Figure 2E); this layering becomes indistinct at caudal levels (Figure 3E). The MeD did not show any layering organization (Figures 2E, 3E).

##### The Anterior Cortical Nucleus (ACo)

The ACo is present in the rostral half of the AC (Figures 1D, 2D, 3D). It limits with structures like the PAC and the NLOT in anterior levels (Figure 1D) and the PCo and Me more caudally (Figures 2D, 3D). The PCo occupies half of the AC starting at more middle rostrocaudal levels than the ACo (Figures 2D, 3D); this nucleus is bordered dorsally by the ACo (Figures 2D, 3D), ventrally by the PAC (Figures 2D, 3D) and laterally by the AB (Figure 2D) or the AHI (Figure 3D). Nissl staining reveals a cell-free layer 1 (L1), a highly packed and darkly stained L2 and a layer 3 (L3) with less packed and also strongly stained neurons (Figure 1E).

##### The Posterior Cortical Nucleus (PCo)

The PCo occupies half of the AC starting at more medium rostrocaudal levels than the ACo (Figures 3D, 4D); this nucleus is bordered dorsally by the ACo (Figures 3D, 4D), ventrally by the PAC (Figures 3D, 4D) and laterally by the AB (Figure 3D) or the AHI (Figure 4D). This nucleus has two layers: L1 contains a few scattered neurons and L2 more densely packed neurons (Figure 3F); AChE staining intensity was higher in L1 (Figure 3C).

##### Nucleus of the Lateral Olfactory Tract (NLOT)

This nucleus is located in the rostral third of the AC, between the ACo and Me. It is divided into two layers characterized by a narrow band of densely packed cells in L2 (Figure 1E) and a high level of AChE staining in L1 (Figure 1C).

##### The Periamygdaloid Cortex (PAC)

The PAC is located in the medial surface of the AC and extends along its rostral half. This area showed a rudimentary organization of three layers that will be described below. In the most rostral level, the PAC is located between the ACo and the entorhinal cortex (E) (Figure 1D) whereas in mid-levels it is bordered by the PCo and the E (Figures 2D, 3D). We subdivided it into oral (PACo), 1 (PAC1), 2 (PAC2), 3 (PAC3) and sulcal subdivisions (PACs):

• *PACo*: this is bordered ventrally by the E and dorsally by PAC1 (Figure 1D). Cells in L2 are densely distributed and show heavy Nissl staining, while L3 contained more sparsely distributed neurons (Figure 1F). PACo shows a moderate AChE staining (Figure 1C).
• *PAC1*: like the PACo, PAC1 shows a moderate AChE staining (Figure 1C), but the L2 is more diffuse than in PACo in Nissl staining (Figure 1F).
• *PAC2*: this subdivision is located between the PCo and PAC3 (Figures 2D, 3D). It contains small, densely packed neurons in L2 (Figure 2F) while L3 has a bit more scattered pale Nissl-stained cells that appear more strongly stained in caudal levels (see asterisk in Figure 3B). The PAC2 has lower AChE (Figures 2C, 3C) and moderate Gallyas staining intensity in L2 and L3 but heavier staining in L1 (Figures 2A, 3A).
• *PAC3*: this region is located between PAC2 and either the E or PACs (Figures 2D, 3D, respectively). It is characterized by an almost cell-free L1 and a more cell-dense L2 which is not clearly separated from L3 (Figures 2G, 3G). PAC3 showed low AChE staining (Figures 2C, 3C), moderate myelin fiber density in L2 and L3 but high fiber density in L1 (Figures 2A, 3A).
• *PACs*: located between PAC3 and the E (Figure 3D), PACs is characterized by a thin layer of neurons in L1 (Figure 3G), and again L2 and L3 without clear delimitation (Figure 3G).

#### The Deep Nuclei

These nuclei occupy most of the AC volume and show a great heterogeneity regarding their cellular morphology and AChE activity. Several myelin fiber bundles separate these deep nuclei, further facilitating their identification. Four deep nuclei were delineated: L, B, AB and Pal.

##### Lateral Nucleus (L)

The L occupies the whole rostrocaudal extension of the AC (Figures 1–5), being bordered dorsally and laterally by myelin structures that continues with the ec ventrally: the inferior fronto-occipital fasciculus (ifo, Figures 2A, 3A), the uncinated fasciculus (unc, Figures 2A, 3A) and the inferior longitudinal fasciculus (ilf, Figures 2A, 3A, 4A). The L is clearly distinguished from the B by its low AChE staining in every anteroposterior level (Figures 1C, 2C, 3C, 4C).

**Figure 6:**
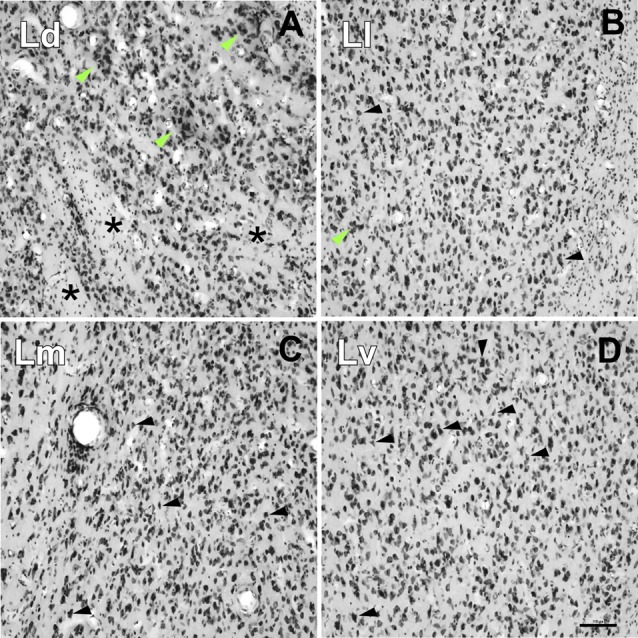
High-power photomicrographs showing the cytoarchitecture of the lateral nucleus subdivisions. **(A)** Ld neurons are medium to large sized and clustered (green arrows). Asterisks indicate accumulations of small areas of white matter. **(B)** Neurons in the Ll have different shapes (black arrows) and are less densely packed than in the Ld **(A)** and Lm **(C)**, and more densely than in the Lv **(D)**; neuron clusters are rare (green arrow). **(C)** Neurons in the Lm have a variety of shapes and sizes (black arrows). **(D)** The Lv is characterized by a relatively low neuronal density with great variability in their sizes and shapes and poorly stained. Scale bar: 100 μm.

Extrapolating the criteria of the Pitkänen and Amaral study (1998), we identified four main divisions within the L: dorsal (Ld), lateral (Ll), medial (Lm) and ventral (Lv), on the basis of cell size and packing density (Figure 6) and on AChE neuropil staining (Figures 2C, 3C, 4C).

• *Dorsal Division* (*Ld*): it is mostly found in the rostral half of the L (Figures 2D, 3D). In Nissl preparations, neuron clusters are frequently visible (see green arrows in Figure 6A). The presence of small areas of accumulated white matter is also characteristic of this subdivision (see asterisks in Figure 6A). This division is not visible at caudal levels (Figures 4D, 5D), where is substituted by the striatum, a structure characterized by a high staining of AChE (Figures 4C, 5C).
• *Lateral Division (Ll)*: it appears at nearly the rostral pole of the AC and extends to the caudal pole of the L. It contains fewer myelin fibers than the Ld (Figures 2A, 3A). Neurons are less densely packed here than in dorsal and medial divisions, but more densely than in the ventral division (Figure 6B).
• *Medial Division (Lm)*: this is present through the whole anteroposterior extension of the L and occupies the most dorsal position of the L in the caudal third of the AC (Figure 4D). In Nissl sections, its neurons have a variety of shapes and sizes (black arrows, Figure 6C). The presence of strongly AChE-stained nuclei intercalated between the dorsal and medial subdivisions is also interesting (Figure 3C).
• *Ventral Division (Lv)*: present in the whole rostrocaudal extension of the AC, the ventral division makes up most of the ventral border of the L (Figures 2D, 3D, 4D). The low neuronal density in Nissl sections is remarkable (Figure 6D); these neurons are heterogeneous in size and shape (black arrows, Figure 6D). The ventral division has the lightest AChE (Figures 2C, 3C) and Gallyas (Figures 2A, 3A, 4A) staining of all the L subdivisions, useful features separating it from the Pal (see black arrows in Figures 2C, 3C and white arrow in Figure 2A).

##### Basal Nucleus (B)

The B is separated from its adjacent nuclei by different myelin tracts; these tracts were the ll and intermediate medullary lamina (li) and separate the B and L, and the B from the AB, respectively (Figures 2A, 3A). The B has the highest level of AChE staining of all the AC nuclei (Figures 1C, 2C, 3C, 4C). Based on the size and Nissl staining intensity of the neurons, this nucleus was divided into four subdivisions: magnocellular (Bmag), intermediate (Bint), parvocellular (Bpv) and magnocellular dorsal (Bmd) subdivisions (Figures 2D, 3D), the last having neurons with the same size as the Bmag but a lower Nissl staining. The Bmd can be distinguished from the amygdalostriatal transition area (Ast) due to the smaller neurons of this area (Supplementary Figure S1). The Bpv shows very few or no myelinated fiber bundles compared to the other three basal nucleus subdivisions (Figures 2A, 3A).

##### Accessory Basal Nucleus (AB)

The AB is the most medial of the deep AC nuclei. It is separated from the B by the li (Figures 2A, 3A). It limits dorsally with the ventral subdivision of the Me (MeV) and the lm in rostral and medium anteroposterior levels (Figures 2A, 3A) and with the stria terminalis (st) in caudal levels (Figure 4A). The inferior longitudinal medial fasciculus (ilmf) separates the AB from the PAC (Figures 2A, 3A) and the cortical nuclei in medium levels of the AC (Figure 2A), and from the Hip in the caudal end of the AB (Figure 4A). We identified three subdivisions on the basis of neuron size and AChE staining intensity: magnocellular (ABmag), parvocellular (ABpv) and ventromedial (ABvm); the ABmag held the biggest cells and showed the highest AChE staining intensity (Figures 2C, 3C); the ABvm showed more packed cells than the ABpv (Figures 2B, 3B) and its AChE staining intensity increased through more caudal levels (Figures 2C, 3C, 4C).

##### The Paralaminar Nucleus (Pal)

The Pal is a narrow band of densely-packed neurons (Figures 2B, 3B) occupying the ventral limit of the AC along its rostral half (Figures 1, 2, 3); the Pal is located ventral to the B and between the L and AB (Figures 1D, 2D, 3D) and especially its limit with the L is demarcated by the higher level of AChE in the Pal compared to the L (see black arrows in Figures 2C, 3C). Dorsally it is distinguished from the B by its low intensity of the AChE (Figures 1C, 2C, 3C) and myelin staining (Figures 2A, 3A).

#### The Central Nucleus (Ce)

The Ce is an elongated cylindrical cell mass visible as a roughly circular nucleus in most of the coronal section levels. It is separated from the B by the ld (Figures 2A, 3A) or the I at caudal levels (Figures 4D, 5D), and remains adjacent to the Ast or the AAA at rostral levels (Figures 1D, 2D, 3D). At more caudal levels, this nucleus is interposed between the tail of the Cd and the Me (Figures 3D, 4D). Following the criteria of (Paxinos et al. (2012)) in macaques, we have divided the Ce into medial (CeM), lateral (CeL) and capsular (CeC) subdivisions (Figures 1D, 2D, 3D, 4D), based on the AChE staining intensity (Figures 1C, 2C, 3C, 4C) and the myelinated bundles that separate the three subdivisions, especially in middle and caudal AC levels (Figures 3A, 4A). The CeL stands out by its low packed neurons (Figure 3B, Supplementary Figure S1) and the lack of AChE staining compared to the other two subdivisions (Figures 2C, 3C, 4C).

#### The Intercalated Nuclei (I)

The I are small cell masses intermingled among the AC nuclei, typically between the deep nuclei and the Ce (Figures 2D, 3D, 4D, 5D) or within the L separating its subdivisions (Figure 3D). As seen in Nissl stained sections, their neurons are small, darkly stained and densely packed in clusters (Figures 2B, 3B, 4B, 5B, Supplementary Figure S1 for high magnification); they also showed high intensity of both AChE (Figures 2C, 3C, 4C, 5C) and myelin staining (Figures 3A, 4A).

#### Transition Areas

The AAA is a region that occupies the rostral third of AC (Figures 1D, 2D) and shows intense AChE staining (Figures 1C, 2C).

The AHI is located in the caudal half of the AC, surrounded by structures as the PCo, the PAC, the lm or the AB (Figure 3A) at rostral levels, and the Hip or the st caudally (Figure 4A). The neurons in this area are densely packed and intensely stained in Nissl preparations (Figures 3B, 4B) but show high (Figure 3C) and moderate (Figure 4C) AChE staining intensity.

### Myeloarchitecture

Myelinated axon tracts around and within the AC are identified according to the nomenclature adopted in macaques (Amaral et al., 1992; Mai et al., 2004; Schmahmann and Pandya, 2010; Mori et al., 2017).

Table 1 summarizes all the tracts identified, as well as their precise location (Figures 1A, 2A, 3A, 4A, 5A). Gallyas staining revealed that the AC contains numerous intra-amygdaloid myelinated fiber bundles. These include a group of small myelin tracts which are lm, li, ll and ld. The ld is a horizontal tract that exits the ec medially, separating the B from the Ce (Figures 2A, 3A); it then originates a descending tract called the li, which separates the B from the AB nuclei (Figures 2A, 3A). The ll leaves the ec in a more ventral direction than the dorsal one, and separates the L from the B. Finally, the lm, the most medial of these tracts, separates the Me from the AB (Figures 2A, 3A); the Me is also bordered dorsally (Figure 1A) and laterally (Figure 2A) by the amygdalo hypothalamic fasciculus (ahf).

**Table 1:** Nomenclature and localization of the myelinic tracts present in the amygdaloid complex (AC).

Tract	Localization
Medial medullary lamina (lm); Mai et al. (2004)	Separates Me from AB and Ce
Lateral medullary lamina (ll); Mai et al. (2004)	Separates L from B
Intermediate medullary lamina (li); Mai et al. (2004)	Separates B from AB
Dorsal medullary lamina (ld) present study	Separates B from Ce
Inferior fronto-occipital fasciculus (ifo); Mori et al. (2017)	Inferior to acp
Uncinated fasciculus (unc); Schmahmann and Pandya (2010) and Mori et al. (2017)	Inferior to ifo
Inferior longitudinal fasciculus (ilf); Schmahmann and Pandya (2010) and Mori et al. (2017)	Inferior to unc
Amygdaloid capsule (amc) present study	Ventral border of AC, inferior to ilf
Sublenticular part of the internal capsule (slic); Mori et al. (2017)	Between the Cd, the Ce and the L (caudal level)
Inferior longitudinal medial fasciculus (ilmf); Mori et al. (2017)	Separates AB from PAC (rostral level) and AB from Hip (caudal level)
Amygdalo hipothalamic fasciculus (ahf)	Dorsal and lateral to the Me

*^5^Avendaño and Reinoso-Suarez (1975). For abbreviations see list for abbreviations*.

Another group of larger white matter tracts surrounding the AC that could be clearly identified in the Gallyas stained sections is composed of the ec (Figures 1A, 2A, 3A), the ifo (Figures 2A, 3A, 4A, 5A), the unc (Figures 1A, 2A, 3A, 4A, 5A) and the ilf (Figures 2A, 3A, 4A, 5A). The ilf continues as the amc at the ventral border of the AC (Figures 2A, 3A, 4A, 5A), and the amc as the ilfm at the medial border of the AC (Figures 2A, 3A, 4A), this last bundle separating the PAC and the AB (Figures 2A, 3A) and the Hip from the AB (Figure 4A). Curiously, the sublenticular part of the internal capsule (slic) maintained a spatially close relationship with the AC in caudal levels (Figure 4A). The st appears in the AC at its caudal pole (Figures 4A, 5A) among the AHI, the Me and the AB. The intraamygdaloid portion of the stria terminalis (STIA) raise from fibers that abundantly pierce the Ce, B and AB nuclei (Figure 4A).

## Discussion

We provide here a detailed parcellation of nuclear cell masses and myelinated fiber tracts of the marmoset AC based on Nissl, AChE and myelin staining patterns. The discussion below focuses on the most salient findings and the similarities or differences between marmoset AC anatomy and published observations in other primates.

The macroscopic resemblance in the global histological layout of marmoset AC to that of humans reflects, beyond phylogenetic proximity, probably also common behavioral demands. Like us, marmosets depend largely on visual discrimination and complex vocalization codes for navigating dangerous, changing and hierarchical social networks, as well as for reacting against predators or foraging over wide territories. On the other hand, despite the gross-anatomical AC resemblance, many key cellular/molecular aspects may well be subtly but crucially different. These aspects may include differences in absolute/relative cell numbers (Herculano-Houzel et al., 2015), in somato-dendritic complexity and signal-carrying capacity (Eyal et al., 2016), in anatomical weight/spatial distribution of the extrinsic and internuclear synaptic connections (Thiebaut de Schotten et al., 2012) as well as species-specific idiosyncrasies in neurotransmitter/hormone receptor subtypes, their subcellular localization, and/or their expression levels (Neumann, 2008; Lefevre et al., 2017; Stetzik et al., 2018).

### The Superficial Group Nuclei Are Relatively Small, as in Other Primates, Yet Very Heterogeneous Histologically

We subdivided the Me into dorsal and ventral parts based on cytoarchitecture and AChE staining. The cytoarchitecture we observed for this nucleus, including a layered organization, when present, is not much different to that of *Macaca fascicularis* (Amaral and Bassett, 1989; Pitkänen and Amaral, 1998). In the macrosmatic rodents, the Me is commonly subdivided into four cytoarchitectonic divisions (Canteras et al., 1995), but the present is the first study to establish subdivisions in this nucleus in primates.

The organization of the anterior and posterior cortical nuclei in marmosets resembles the one observed in other primates (Amaral and Bassett, 1989; Amaral et al., 1992; Pitkänen and Amaral, 1998). Studies in mice have shown that the cortical amygdaloid nuclei mediate the generation of innate odor-driven behaviors. For example, different cell populations in the cortical nuclei are capable of eliciting innate responses to either appetitive or neutral odors; neurons responsive to appetitive behavior were concentrated in the posterolateral cortical amygdala whereas those responding to neutral stimuli were located in the anterior cortical amygdala (Root et al., 2014). This segregation could be related to the existence of highly topographic projections from the olfactory glomeruli to the cortical nuclei in mice (Sosulski et al., 2011) and primates (Carmichael et al., 1994; Liebetanz et al., 2002).

The NLOT of marmosets is relatively small, as in other in primates (Amaral et al., 1992), compared to macrosmatic species such as cats and rats (Krettek and Price, 1977). The PAC is a heterogeneous region covering much of the medial surface of the AC. The parcellation and nomenclature referring to this region is inconsistent between previous studies. We adopted the same five subdivisions used for *Macaca* *fascicularis* by Carmichael et al. (1994) and Pitkänen and Amaral (1998). The olfactory projections reaching the marmoset PAC have been shown to be comparable to those in other microsmatic primates (Liebetanz et al., 2002).

### The Global Arrangement and Subdivisions of the Deep Group of AC Nuclei Resembles That of Other Primates, Including Humans

The Deep group of AC nuclei is relatively much bigger in humans than in New or Old World monkeys. However, the overall organization and the parcellation of these Deep nuclei in marmosets are similar to other primates.

The L is the largest nucleus of the primate AC. Studies of the L in rats, monkeys and humans suggest many basic similarities in the organization of its chemoarchitecture and connectivity (Pitkänen and Kemppainen, 2002). Published marmoset studies (Roberts et al., 2007; Paxinos et al., 2012) did not attempt a parcellation of this nucleus. Based on differences in AChE staining in the *Macaca fascicularis* AC, Price et al. (1987) and Amaral and Bassett (1989) distinguished two main subdivisions (ventrolateral and dorsomedial) within the L. In a subsequent study in the same species (Pitkänen and Amaral, 1998) four subdivisions were proposed: dorsal, dorsal intermediate, ventral intermediate and ventral subdivisions. Equivalent subdivisions have been proposed for the human AC, albeit with a different nomenclature: (external, dorsal, lateral and medial; Sims and Williams, 1990; García-Amado and Prensa, 2012).

The neural connectivity of L in macaques is relatively well documented. The insular (Amaral and Insausti, 1992), superior temporal, auditory cortical areas (Yukie, 2002; de la Mothe et al., 2006), as well as the inferotemporal high level processing visual cortical areas (Aggleton et al., 1980; Iwai and Yukie, 1987; Ghashghaei and Barbas, 2002; Kravitz et al., 2013) have been shown to send projections mainly to dorsal portions of the nucleus. In marmosets, the connections between the auditory cortex and the L have been reported to be bilateral and are also restricted to specific dorsal portions of this nucleus (see Figures 4B, 5 in Reser et al., 2009). The presence of intranuclear connections from the dorsal to ventral zones of L suggest that information processing may be to some extent compartmentalized or sequential across the various L subdivisions (Pitkänen and Amaral, 1998). The finding in macaques that sensory or association thalamic nuclei such as the medial pulvinar send inputs to dorsal regions of the L (Jones and Burton, 1976; Day-Brown et al., 2010) is consistent with the above view.

In rats, in addition to sensory or association thalamic nuclei projecting to the dorsolateral division, some midline nuclei (paratenial and central medial) project to the ventromedial division of the L (Turner and Herkenham, 1991). These data hint at the possibility that two separate thalamic systems innervate different divisions of the L. An additional indication of the functional heterogeneity of the various subdivision of L is that, in rats, levels of c-Fos expression during fear conditioning acquisition, specifically in response to the foot-shock unconditioned stimulus are clearly different in the dorsal and ventral parts of L (Lanuza et al., 2008).

Differences in cell soma size, packing density of AChE neuropil staining, allow the consistent identification of four divisions (Bmag, Bint, Bpv and Bmd) within the B. In the present study we distinguish a subdivision of the B (the Bmd), which corresponds to the dorsolateral subdivision described in Paxinos et al. (2012). In marmosets, the cytoarchitecture of the Bmd is clearly different from that of the Bmag (see Supplementary Figure S1). An equivalent subdivision was not made in previous Old World (macaques: Pitkänen and Amaral, 1998; Cavada et al., 2000; Carlo et al., 2009; humans: García-Amado and Prensa, 2012) and New World (*Cebus apella*, Carlo et al., 2009; marmosets, Roberts et al., 2007; *Saimiri sciureus*, Brady et al., 1992) primate studies.

The tract-tracing study of Roberts et al. (2007) in marmosets has shown that, as in macaques (Carmichael and Price, 1995; Ghashghaei et al., 2007) the B nucleus is reciprocally connected with prefrontal cortical areas in a topographic fashion: medial prefrontal areas are preferentially connected with ventral portions of the B whereas the lateral prefrontal areas project to more dorsal parts of this nucleus. It has been proposed that prefrontal connections with B provide the predator-related attentional and motivational cues involved in fear responses (Bindi et al., 2018).

We distinguish three divisions (magnocellular, parvocellular and ventromedial) within in the marmoset AB, following the criteria of Price et al. (1987), Pitkänen and Amaral (1998) and Pitkänen and Kemppainen (2002) in *Macaca fascicularis*. Similar divisions, albeit labeled with a somewhat different nomenclature were noted by Cavada et al. (2000) in *Macaca nemestrina*. In contrast, other studies divided AB in only magnocellular and parvocellular divisions (Carlo et al., 2009, *Cebus apella and Macaca mulatta*; Brady et al., 1992, *Saimiri sciureus*; Roberts et al., 2007, *Callithrix jacchus*). Studies in rats indicate that the AB receives direct projections containing predator odor information from the medial amygdala (Bindi et al., 2018).

As in other primates, the marmoset Pal is an elongated lamina of cells extended along the ventral border of the B, ventrally surrounded by the amc. As in other non-human primates, we did not distinguish subdivisions in this nucleus (Pitkänen and Amaral, 1998; Cavada et al., 2000; Carlo et al., 2009). However, in humans the Pal has been subdivided in medial and lateral portions, based on cytoarchitecture (Pitkänen and Kemppainen, 2002). Interestingly, the Pal is not apparent in non-primates such as rats and cats (Krettek and Price, 1978). The macaque Pal contains a dense concentration of small cells, many of which reportedly have immature features; this has led to the suggestion that this nucleus may have a particular role in plasticity mechanisms (deCampo and Fudge, 2012).

### The Central Nucleus and the Intercalated Nuclei of Marmosets Closely Resemble Those of Other Primates

We distinguish three subdivisions (medial, lateral and capsular) in the Ce. This organization is equivalent to that described in macaques by Freedman and Shi (2001) and marmoset (Paxinos et al., 2012). Other primate studies distinguished only medial and lateral subdivisions (Amaral et al., 1992; Brady et al., 1992; Cavada et al., 2000; Cho and Fudge, 2010). In humans, five Ce subdivisions have been distinguished (Sims and Williams, 1990).

Previous studies in macaques (Amaral et al., 1992; Pitkänen and Amaral, 1998) and humans (Sorvari et al., 1995) observed small-celled clusters equivalent in appearance and relative position to the I nuclei we describe. In a recent macaque study, Zikopoulos et al. (2016) concluded that the I are not isolated cell clusters but, rather, a neuronal net paucisynaptically connecting with each other the deep, Me and Ce nuclei. Based on its AChE staining pattern it is plausible that the CeC is equivalent to the capsular division of the Ce in rats (McDonald, 1992; Pitkänen and Amaral, 1998).

## Myelin Fiber Tracts Within and Around AC

As in macaques (Johnston, 1923; Amaral et al., 1992; Schmahmann and Pandya, 2010), the major fiber tracts around and within the marmoset AC can be consistently identified using myelin stains and are helpful for nuclei delimitation. In addition, we identify the ld and the ahf, which had been described in cats, but not in primates (Avendaño and Reinoso-Suarez, 1975; Krettek and Price, 1978; Amaral et al., 1992). The location of each myelin tract is summarized in Table 1.

The myelinated fiber bundles or isolated fibers fanning through some AC nuclei may correspond, to a large extent, to long-range axonal projections leaving and/or reaching these nuclei. The Ld is also characterized by being traversed by a large number of fibers organized in bundles, which help to delimit it (Figures 2A, 3A). This subdivision of the L is the one that receives most of the information reaching the AC, mainly from cortical areas, but also from the thalamus (see above); from this subdivision, the information flows to the other L subdivisions (Pitkänen and Amaral, 1998).

The B contains a particularly large number of such fibers (Figures 3A, 4A). This nucleus is known to innervate the ventromedial striatum (Fudge et al., 2002), and is richly interconnected with the prefrontal, orbitofrontal, anterior cingulate cortices (Carmichael and Price, 1995; Cavada et al., 2000; Roberts et al., 2007). A recent study of B projections to vision-related areas in the temporal and occipital lobes showed that fibers originating in dorsal regions of this nucleus do not form a tract when they leave the AC, but rather form scattered bundles in the B, bundles that exit through the L or Pal nuclei and have been named the “temporal-occipital amygdalo-cortical pathway” (Freese and Amaral, 2005).

The ifo, ilf, imlf and unc fasciculi, as well as the amc together, correspond to the classical “ventral bundle” (see Table 2, Mori et al., 2017). Studies in primates (Freese and Amaral, 2005) and humans (Mai et al., 2004; Mori et al., 2017) indicate that these tracts mainly contain fibers reciprocally connecting the AC with the temporal cortex. The unc, for example, is a conduit for axons extended between the inferotemporal and the superior temporal areas and ventrolateral areas of the prefrontal cortex (Schmahmann and Pandya, 2010).

**Table 2:** Comparison between the nomenclatures adopted for AC white matter structures in classic studies (Johnston, 1923; Amaral et al., 1992) and our study.

Classical	Our study	Localization
Lateral bundle	ll	Between L from B
Intermediate bundle	li	Separates B from AB
Medial bundle	lm	Separates Me, Ce and AB
Ventral bundle*	ifo, unc, amc, ilf and ilmf	Lateral and ventral border of AC

*The ventral bundle was subdivided into more portions based on its location and functional studies. For abbreviations see list for abbreviations.

Diffusion tractography magnetic resonance imaging (DTI-MRI) can create virtual visualizations of white matter tracts of increasing spatial resolution. A recent *in vivo* MRI study in marmosets was, in fact, able to identify the major association tracts (unc, ifo and ilf) and noted the surprising resemblance of their overall arrangement to that in humans (Schaeffer et al., 2017). Anomalous (hypo or hyper) connectivity of the AC in circuits that encompass cognition, emotion regulation, and sensorimotor processes are observed in depression and anxiety disorders (Klumpp et al., 2018). There is also abundant evidence of specific impairment of the AC surrounding myelin tracts in schizophrenia and bipolar disorder (Mori et al., 2017; Schaeffer et al., 2017; Shiba et al., 2017). The description of AC myelin tracts in the present study may thus be of interest for future *in vivo* investigations of AC anatomy and function using DTI-MRN in marmosets.

## Author Contributions

RL, PAGM and AC-C perfused the animals and performed the neurohistological processing with support from JSC and EN. PAGM, MG-A and FC performed the analysis and delimitation of the amygdaloid nuclei. MG-A, PAGM and FC wrote the article with input from rest of the authors.

## Conflict of Interest Statement

The authors declare that the research was conducted in the absence of any commercial or financial relationships that could be construed as a potential conflict of interest.

## Footnotes

^1^https://brainminds.jp/en/

## Supplementary Material

FIGURE S1 High-power photomicrographs showing the cytoarchitecture of the Bmd and adjacent areas. The Bmd is distinguished by its fewer Nissl-stained neurons compared to the Bmag; also the density of neurons seems to be slightly lower in the Bmd. It is separated from the Ast by one of the I. Note also differences among the cytoarchitecture of the three Ce subdivisions, showing that the CeL has a lower density of cells than the CeM and the CeC. Scale bar: 250 μm.

## Abbreviations

AAA: anterior amygdaloid area
AB: accessory basal nucleus
ABmag: magnocellular subdivision of the accessory basal nucleus
ABpv: parvocellular subdivision of the accessory basal nucleus
ABvm: ventromedial subdivision of the accessory basal nucleus
ahf: amygdalo hypothalamic fasciculus
AHI: amygdalohippocampal area
acp: posterior limb of the anterior commissure
AC: amygdaloid complex
ACo: anterior cortical nucleus
amc: amygdaloid capsule
Ast: amygdalostriatal transition area
B: basal nucleus
Bmd: dorsal magnocellular subdivision of the basal nucleus
Bint: intermediate subdivision of the basal nucleus
Bmag: magnocellular subdivision of the basal nucleus
Bpv: parvocellular subdivision of the basal nucleus
Cd: caudate nucleus
Ce: central nucleus
CeC: capsular subdivision of the central nucleus
CeL: lateral subdivision of the central nucleus
CeM: medial subdivision of the central nucleus
Cl: claustrum
E: entorhinal cortex
ec: external capsule
DEn: dorsal subdivision of the endopiriform nucleus
ex: extreme capsule
Hip: hippocampus
I: intercalated nuclei
ifo: inferior fronto-occipital fasciculus
ilf: inferior longitudinal fasciculus
ilmf: inferior longitudinal medial fasciculus
L: lateral nucleus
L1: Layer 1
L2: Layer 2
L3: Layer 3ld: dorsal medullary lamina
Ld: dorsal subdivision of the lateral nucleus
li: intermediate medullary lamina
ll: lateral medullary lamina
Ll: lateral subdivision of the lateral nucleus
lm: medial medullary lamina
Lm: medial subdivision of the lateral nucleus
Lv: ventral subdivision of the lateral nucleus
Me: medial nucleus
MeD: dorsal subdivision of the medial nucleus
MeV: ventral subdivision of the medial nucleus
NLOT: nucleus of the lateral olfactory tract
opt: optic tract
PAC: Periamygdaloid cortex
PACo: Periamygdaloid cortex, oral subdivision
PACs: Periamygdaloid cortex, sulcal subdivision
PAC1: Periamygdaloid cortex, subdivision 1
PAC2: Periamygdaloid cortex, subdivision 2
PAC3: Periamygdaloid cortex, subdivision 3
Pal: paralaminar nucleus
PCo: posterior cortical nucleus
rf: rhinal fissure
SI: substantia innominata
slic: sublenticular part of the internal capsule
st: stria terminalis
STIA: intraamygdaloid portion of stria terminalis
unc: uncinated fasciculus.
