# Supplementary figures and images for: Cyto- and Myelo-Architecture of the Amygdaloid Complex of the Common Marmoset Monkey (Callithrix jacchus)
Source: Front Neuroanat. 2019 Mar 27;13:36. doi: 10.3389/fnana.2019.00036 (PMC6446959; doi:10.3389/fnana.2019.00036)

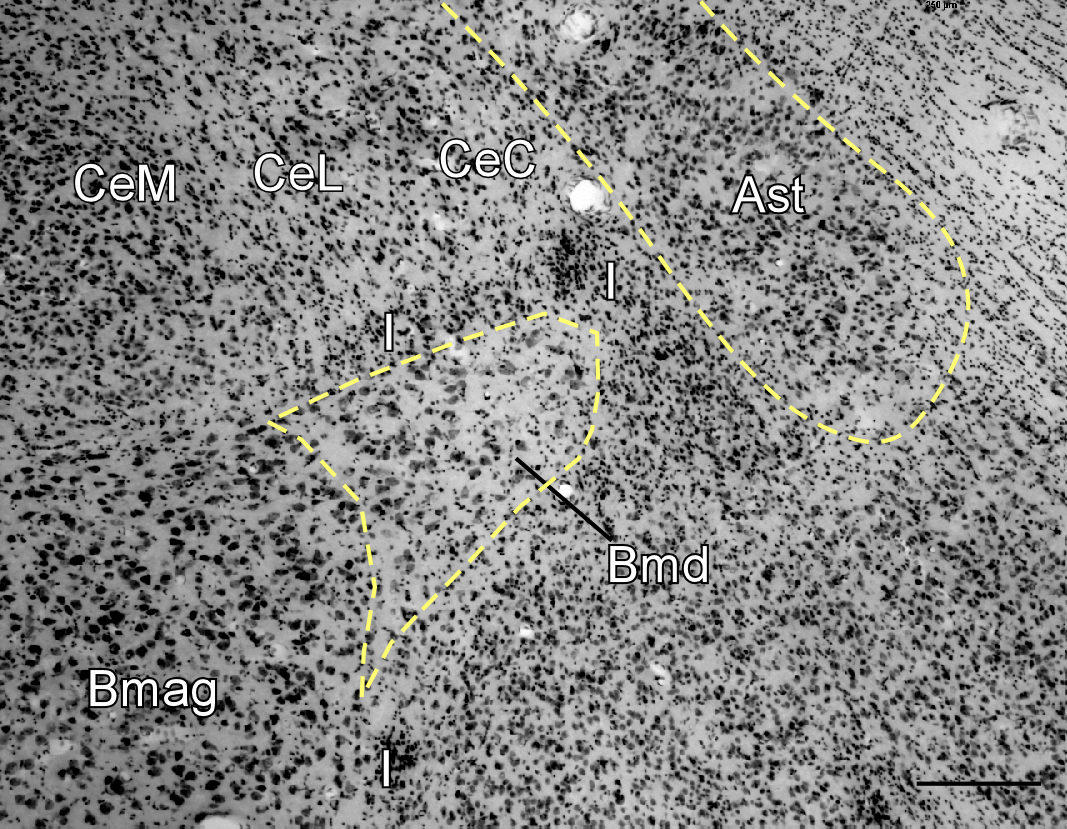

Supplement: FIGURE S1 — High-power photomicrographs showing the cytoarchitecture of the Bmd and adjacent areas. The Bmd is distinguished by its fewer Nissl-stained neurons compared to the Bmag; also the density of neurons seems to be slightly lower in the Bmd. It is separated from the Ast by one of the I. Note also differences among the cytoarchitecture of the three Ce subdivisions, showing that the CeL has a lower density of cells than the CeM and the CeC. Scale bar: 250 μm. [file Image_1.TIF]
